# Effect of pretreatment with transdermal testosterone on poor ovarian responders undergoing IVF/ICSI: A meta-analysis

**DOI:** 10.3892/etm.2014.1683

**Published:** 2014-04-14

**Authors:** SHAN LUO, SHANGWEI LI, XIAOHONG LI, LANG QIN, SONG JIN

**Affiliations:** Reproductive Medical Center, Department of Obstetrics and Gynecology, West China Second University Hospital of Sichuan University, Chengdu, Sichuan 610041, P.R. China

**Keywords:** androgens, *in vitro* fertilization, poor ovarian responders, randomized controlled trials, testosterone

## Abstract

In order to identify and describe the effectiveness of transdermal testosterone pretreatment on poor ovarian responders, MEDLINE, EMBASE, the Cochrane library and the Chinese biomedical database were searched for randomized controlled trials (RCTs). Three RCTs, which compared the outcomes of female pretreatment with transdermal testosterone prior to *in vitro* fertilization/intracytoplasmic sperm injection (IVF/ICSI) with those of control groups, were included in the present review. The three RCTs enrolled a total of 221 randomized subjects. The meta-analysis revealed that females who received transdermal testosterone treatment prior to their IVF/ICSI cycle had a two-fold increase in live birth rate [risk ratio (RR)=2.01, 95% confidence interval (CI) 1.03–3.91], clinical pregnancy rate (RR=2.09, 95% CI 1.14–3.81) and a significantly more oocyte retrieved [mean difference (MD)=1.36, 95% CI 0.82–1.90]. The current findings provide evidence that pretreatment with transdermal testosterone may improve the clinical outcomes for poor ovarian responders undergoing IVF/ICSI. However, the results should be interpreted with caution due to the small sample size of the studies used and the heterogeneities. Further good quality RCTs would be needed to reach further conclusions.

## Introduction

Poor ovarian response to controlled ovarian hyperstimulation (COH) remains a major problem in assisted reproduction. Although no clear definition has been agreed, poor ovarian response in the context of *in vitro* fertilization (IVF) treatment is usually defined as failure to achieve ≥3 oocytes or a certain estradiol concentration in response to ovarian stimulation. The incidence in a population undergoing assisted reproduction treatment is estimated to be 9–24% (1,2). Over the years, numerous techniques and therapies have been developed in an effort to improve success in the poor responders, but few have met with success (3,4)

Androgens and their ovarian receptors have been shown to play an important role in ovarian physiology and follicular growth by numerous studies (5–7). Recently, clinicians have attempted to improve the ovarian response in poor ovarian responders by using androgens or androgen modulators prior to *in vitro* fertilization/intracytoplasmic sperm injection (IVF/ICSI) treatment. The main androgen treatments involve direct androgen supplementation [including dehydroepiandrosterone (DHEA) and testosterone] and the indirect increase of intra-ovarian androgen levels. The latter has been achieved through aromatase inhibitors (anastrozole and letrozole), which are able to elevate intra-ovarian androgen levels by blocking the conversion of androgen substrate to estrogen (8). Among the androgens and androgen modulators mentioned above, transdermal testosterone treatment is most attractive due to its easy application, convenience, painlessness to the patient and most importantly its safety as, in contrast to oral androgen treatment, androgen is not first metabolized in the liver. The aim of the present study was to review the current studies available on the use of transdermal testosterone in poor ovarian responders undergoing IVF/ICSI.

## Materials and methods

### Literature search

MEDLINE, EMBASE and the Cochrane library were searched with no time limit applied to any database. To identify relevant studies, a combination of medical subject headings (MeSH) and text words were used as follows: i) poor ovarian response (‘poor response’, ‘low response’ or ‘bad response’); ii) IVF (‘*in vitro* fertilization’ or ‘IVF’, or ‘intracytoplasmic sperm injection’ or ‘ICSI’); iii) COH (‘controlled ovarian hyperstimulation’ or ‘ovarian stimulation’); and iv) testosterone (‘androgen’ or ‘testosterone’). These key words were combined using ‘AND’ to search for studies relevant to the subject under investigation. The Chinese Biomedical Literature Database (CBM) was also searched using the same keywords in the Chinese language. In addition, the reference lists of the studies to be included were individually searched in order to identify other potentially eligible studies. Studies published in languages other than English and Chinese were not considered. The study selection was undertaken by two of the authors (Xiaohong Li and Lang Qin).

### Study selection and data extraction

Studies that were randomized controlled trials (RCTs) and which compared the clinical outcomes of the pretreatment of poor ovarian responders, with or without transdermal testosterone, prior to the IVF/ICSI cycle were selected. No pre-specified definition for low response was used for the search strategy in order to include all potentially relevant published studies and capture the full range of diagnostic criteria. Studies were selected in two steps. First, the titles and abstracts from the electronic searches were analyzed independently by the two reviewers. Full papers of all citations that were likely to meet the predefined selection criteria were also obtained. In the second step, the final decisions of whether to include or exclude the study were made upon examination of the full manuscript. If there were duplicate studies, the study that reported more detailed data was included for analysis.

The two reviewers completed the data extraction and quality assessment. Selected studies were evaluated for methodological quality using standard forms adapted from the Cochrane Handbook for Systematic Reviews of Interventions (9) and the quality of reporting of meta-analyses checklist (10). When necessary, the reviewers wrote to the authors and obtained extra information and raw data. Outcome data were extracted from each study in 2×2 tables.

### Definition of outcomes measured

Outcomes measured included: i) the live birth rate; ii) the clinical pregnancy rate; iii) the number of oocytes retrieved; iv) total follicle-stimulating hormone (FSH) dose and total duration of ovarian stimulation; v) the cancellation rate; and vi) any adverse effects of testosterone. The rates of live birth, clinical pregnancy and cancellation were calculated on the basis of the number of initiated cycles.

### Risk of bias in individual studies

To ascertain the validity of each study, the selected trials were assessed for their methodological quality on the basis of information reported in the original published papers. The study quality was analyzed by checking the adequacy of randomization, comparability of the groups at baseline (examining baseline characteristics for any substantial differences), the use of blinding, the completeness of the follow-up, and the reliability with a priori sample size estimation. The level of evidence for primary research was evaluated in five grades (11): level 1, high-quality RCT with either statistically significant differences or no statistically significant differences but with narrow confidence intervals; level 2, lesser quality RCT (e.g. <80% follow-up, no blinding, or improper randomization), or prospective comparative study; level 3, case control study or retrospective comparative study; level 4, case series; level 5, expert opinion.

### Statistical analysis

Dichotomous outcomes were expressed as a risk ratio (RR) with 95% confidence interval (CI) using a fixed effects model. Continuous outcomes were expressed as the mean difference (MD) with 95% CI. The treatment effects were statistically evaluated using the I^2^ statistic to quantify heterogeneity across studies (12). Statistical analyses were performed using RevMan 5.0 software (Cochrane Collaboration, Oxford, UK).

## Results

### Identification of articles

**Figure 1:**
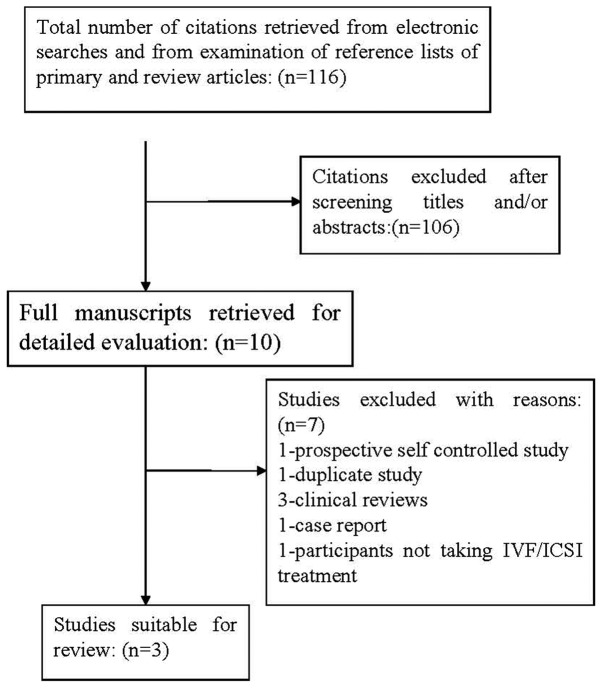
Flow chart of study selection. IVF/ICSI, *in vitro* fertilization/intracytoplasmic sperm injection.

The combined searches identified 116 studies for review. The titles and abstracts of the citations were cross-checked and duplicates were removed by the investigators independently. Studies were removed at initial screening based on the title or abstract if it could be determined that the publication did not meet the inclusion criteria. Ten manuscripts were selected during the initial screening. By examination of the full text, seven studies were excluded: one was a prospective self-controlled study (13), one was a duplicate study (14,15), three were clinical reviews (16–18), one was a case report (19) and one included females not undergoing IVF/ICSI (20). The flow chart for selection of the studies included is given in Fig. 1.

### Data synthesis

From the identified studies, three RCT studies were deemed suitable for review (14,21,22). The main characteristics of the three studies are presented in Table I. These include: ‘poor ovarian responder’ inclusion and exclusion criteria, baseline characteristics, comparison groups, transdermal testosterone dosage and duration, COH protocol and clinical outcomes. Baseline results for a total of 221 participants were included across all studies, with the number of participants in a single study ranging from 49 to 110 individuals. All three studies recruited females with a history of poor ovarian response in previous IVF/ICSI cycles. The mean age of participants was 36–37 years. The main type of transdermal testosterone used was a transdermal testosterone gel or patch, which was applied prior to the COH. All three studies reported all the above outcomes.

### Live birth rate

**Figure 2:**
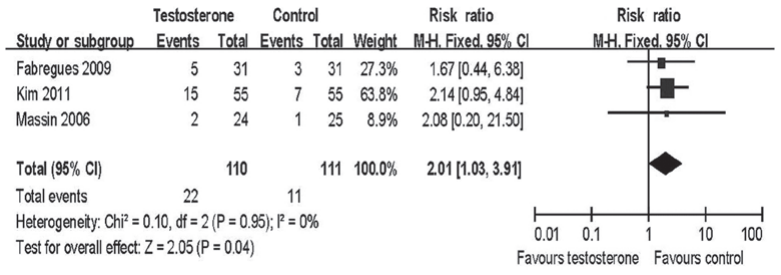
Meta-analysis of transdermal testosterone pretreatment groups vs. the control groups for live birth rate per cycle initiated in poor ovarian responders undergoing *in vitro* fertilization treatment. CI, confidence interval.

The live birth rate per initiated cycle was the index of most interest among the outcomes of IVF/ICSI treatment. All three studies reported this result and a meta-analysis of these studies revealed a significant improvement in the testosterone group compared with the control group [risk ratio (RR)=2.01, 95% confidence interval (CI) 1.03–3.91; Fig. 2].

### Clinical pregnancy rate

**Figure 3:**
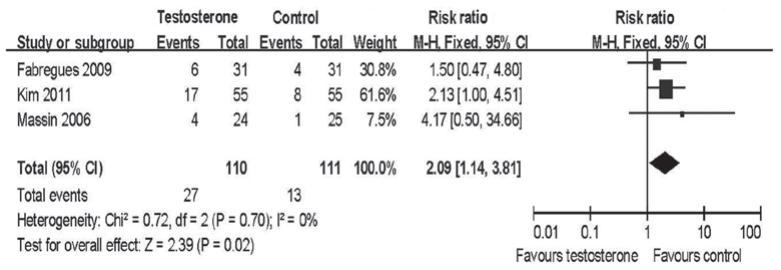
Meta-analysis of transdermal testosterone pretreatment groups vs. the control groups for clinical pregnancy rate per cycle initiated in poor ovarian responders undergoing *in vitro* fertilization treatment. CI, confidence interval.

Meta-analysis of the three RCTs revealed that the clinical pregnancy rate per initiated cycle (RR=2.09, 95% CI 1.14–3.81; Fig. 3) and per cycle transferred (RR=1.97, 95% CI 1.10–3.52; data not shown) in the transdermal testosterone groups was significantly higher than in the control groups.

### Number of oocytes retrieved

**Figure 4:**
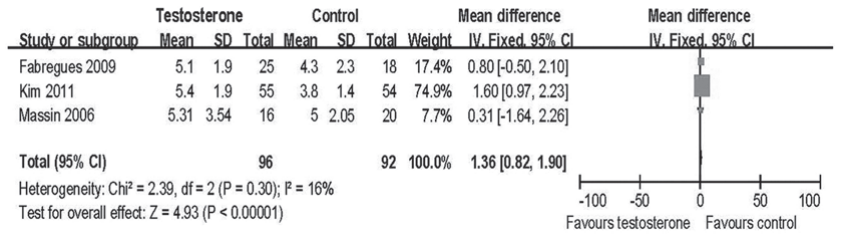
Meta-analysis of transdermal testosterone pretreatment groups vs. the control groups for number of oocytes retrieved in poor ovarian responders undergoing *in vitro* fertilization treatment. CI, confidence interval.

Low ovarian responders were characterized as those having ≤3 oocytes retrieved despite the use of a high gonadotropin dose in previous failed IVF/ICSI cycles. They often faced the risk of cycle cancellation due to fewer developing follicles following COH. Thus, the number of oocytes retrieved was the most direct index used to evaluate the ovarian response to gonadotropin stimulation. All three studies reported a change in the number of oocytes retrieved and meta-analysis revealed that more oocytes were retrieved from the testosterone pretreatment group than from the control group [mean difference (MD)=1.36, 95% CI 0.82–1.90; Fig. 4].

### Total dose of FSH and total duration of FSH stimulation

**Figure 5:**
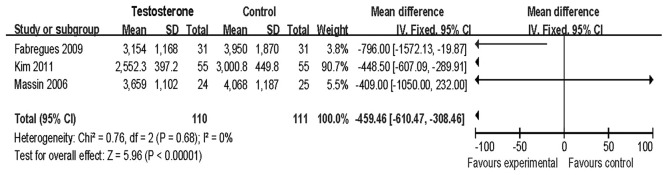
Meta-analysis of transdermal testosterone pretreatment groups vs. the control groups for total FSH dose used in poor ovarian responders undergoing *in vitro* fertilization treatment. CI, confidence interval.

**Figure 6:**
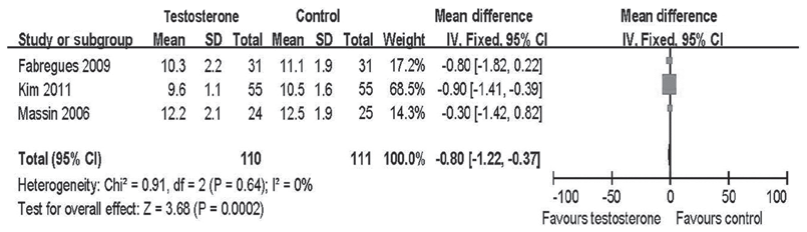
Meta-analysis of transdermal testosterone pretreatment groups vs. the control groups for the total duration of FSH stimulation in poor ovarian responders undergoing *in vitro* fertilization treatment. CI, confidence interval.

The total dose of FSH used and the duration of ovarian stimulation were also effective indices used to evaluate ovarian response. A higher dose of gonadotropins or dosing over a longer period of time suggested a poorer ovarian response. A significantly increased FSH dose and duration were found in the control group compared with the treatment group [(MD=−459.46, 95% CI −610.47 to −308.46; Fig. 5) and (MD=−0.80, 95% CI −1.22 to −0.37; Fig. 6), respectively].

### Cancellation rate

**Figure 7:**
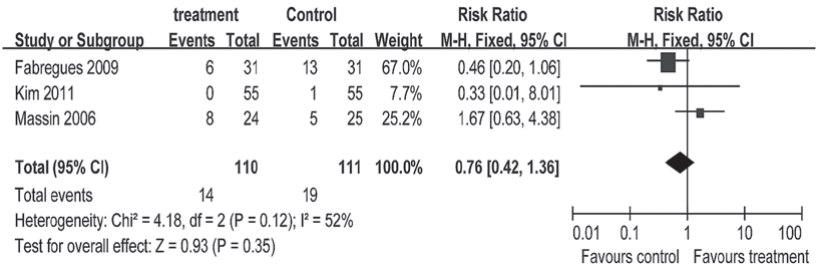
Meta-analysis of transdermal testosterone pretreatment groups vs. the control groups for the cycle cancellation rate in poor ovarian responders undergoing *in vitro* fertilization treatment. CI, confidence interval.

Poor ovarian responders often face an increased risk of cycle cancellation. Meta-analysis revealed no significant difference in cycle cancellation rates between the treatment group and the control group (RR=0.76, 95% CI 0.42–1.36; Fig. 7). However, the criteria for the human chorionic gonadotropin (HCG) injection varied greatly between the studies. In the study by Massin *et al*, only those cycles with at least three follicles reaching 17 mm in diameter could be retrieved. This criteria for oocyte retrieval was more rigorous than in the other two studies and resulted in a very high cycle cancellation rate. This increased the weight of the study by Massin *et al* in the meta-analysis and may have interfered with the final results.

### Testosterone adverse effects

No systematic or local adverse effects attributed to the use of testosterone were reported in any of the three studies.

### Study quality, heterogeneity and compliance

Table II summarizes the study quality information across the three studies, including a brief summary of the strengths and weaknesses of each study. All three studies described the randomization methods used, two by computer generated sequencing (21,22) and one by a randomization list (14). One study was double-blinded (14). Lack of blinding to the investigators in the other two studies gave a moderate risk of performance and detection bias in adjusting FSH dose and evaluating subjective outcomes, for example, the number of high quality embryos and embryos suitable for transfer. All three studies were single-center studies. This avoided any performance bias caused by subtle differences in clinical and IVF/ICSI laboratory techniques among the different centers. However, the time frame of the studies varied from 10 months to 4 years. Studies with a longer time frame may have incurred bias originating from technical improvements in IVF/ICSI laboratory techniques.

## Discussion

Pretreatment of poor ovarian responders with androgen or androgen-modulating agents has been a popular topic for debate for many years, with one other systematic review already published in this area. In that review (17), the author included studies which compared the clinical outcomes between the control group and the androgen (DHEA or testosterone) or androgen-modulating agent (letrozole) pretreatment group. The results of the meta-analysis did not reveal any significant differences in the number of oocytes retrieved and the ongoing pregnancy/live birth rate with androgen supplementation or modulation compared with the control group. Thus, the authors concluded that there was insufficient evidence to support the use of androgen supplementation or modulation to improve live birth outcome in poor ovarian responders undergoing IVF/ICSI treatment. However, due to the lack of high quality studies available in this area at the time, the review had to use the data of trials using different types of androgens. Therefore, the heterogeneity of the including studies, especially the heterogeneity in androgen supplementation protocol (including different types, dosage and duration of androgen used), may have weakened the confidence of the meta-analysis results to some degree. Based on this consideration, the present study analyzed the effectiveness of only one type of androgen on poor ovarian responders. Transdermal testosterone has been considered an ideal type of androgen to use in treatment due to its safety (no supraphysiological concentrations of the steroid are released into the body), easy application, convenience and painlessness to the patient. Several studies (13,14,21,22) have compared the clinical effects of poor ovarian responders with or without transdermal testosterone pretreatment. The present systematic review aimed to gather the existing evidence to explore the effect of transdermal testosterone on poor ovarian responders undergoing IVF/ICSI.

Experimental studies (5–7) demonstrated that treatment with testosterone increased FSH receptor expression in granulosa cells, promoting the initiation of primordial follicle growth and improving the number of growing preantral and small antral follicles in rhesus monkeys. These studies suggested that androgen treatment may amplify the effects of FSH in the ovaries. Barbieri *et al* (23) evaluated the baseline serum testosterone levels of 425 females undergoing IVF and observed that testosterone levels decreased significantly with advancing age. There was also a positive correlation between serum testosterone level and the number of oocytes retrieved. Frattarelli *et al* (24) reported that females with higher baseline levels of testosterone required a lower FSH dose, a shorter duration of ovarian stimulation and were more likely to achieve a pregnancy than females with lower testosterone levels. Consistent with these results, the current systematic review revealed that pretreatment with testosterone in poor ovarian responders undergoing IVF/ICSI significantly increased their oocyte number (MD=1.36, 95% CI 0.82–1.90) and achieve a two-fold improvement in live birth rate per initiated cycle (RR=2.01, 95% CI 1.03–3.91) and clinical pregnancy rate per cycle (RR=2.09, 95% CI 1.14–3.81) compared with the control group. Poor ovarian responders taking IVF/ICSI treatment often face a high risk of cycle cancellation due to few oocytes or good quality embryos being obtained. Thus, the number of oocytes retrieved, clinical pregnancy rate and the live birth rate per cycle initiated were the primary outcomes to be considered when evaluating the curative effect of any treatment. The current study demonstrated that pretreatment with transdermal testosterone was able to effectively improve the clinical outcomes of poor ovarian responders. At the same time, a lower dose of FSH (MD=−459.46, 95% CI −610.47 to −308.46) and a shorter duration of FSH (MD=−0.80, 95% CI −1.22 to −0.37) used were found in the transdermal testosterone group compared with the control group. The results confirmed the effectiveness of transdermal testosterone in improving the ovarian reactivity of poor ovarian responders.

The current systematic review provided evidence that pretreatment with transdermal testosterone prior to IVF/ICSI cycles may improve the clinical outcomes of poor ovarian responders. The validity of the present review relied on its methodological rigor and the components of the primary studies. Great effort and an appropriate protocol were used to gather the evidence. Two independent reviewers extracted data and evaluated the quality of the studies. The level of agreement between the two reviewers was high.

A major problem frequently encountered when selecting studies for meta-analysis is the clinical and methodological heterogeneity of the studies. For the three studies included in the current review, the characteristics used in the studies, such as the definitions for poor ovarian response and the mean ages of the participants, had relatively good homogeneity. However, heterogeneity was still encountered including: the dose and duration of the transdermal testosterone used, the ovarian stimulation protocol, and certain important differences in the quality features between the studies. In addition, the small sample size was another limitation. Thus, the results of the current review should be interpreted with caution. Further good quality RCTs are required to provide more robust evidence and ensure more reliable conclusions in the future.

## Figures and Tables

**Table I:** Summary of study characteristics and clinical outcomes.

Study (Ref.)	Design	Inclusion and exclusion criteria (definition of poor responder)	Participants	Testosterone protocol	COH protocol	Outcomes reported
Kim *et al* (21)	RCT	Inclusion: Low responder defined as <3 oocytes retrieved in the first IVF/ICSI cycle Exclusion: History of endocrine disorder	N=110 Testosterone (n=55), age=37.8±3.0 Control (n=55), age=37.9±2.9	Case: transdermal testosterone gel 12.5 mg/day (1.25 mg/day nominal delivery rate of testosterone) for 21 days preceding the ovarian stimulation Control: none	Case: GnRH antagonist protocol Control: GnRH antagonist protocol	Live birth rate Clinical pregnancy rate No. of oocytes Total dose of FSH Duration of FSH used Cancellation rate Adverse effects
Fábregues *et al* (22)	RCT	Inclusion: History of the first cycle cancelled due to poor follicular response Exclusion: Age >40 years History of previous ovarian surgery, ovarian endometriosis and endocrine and metabolic disorders	N=62 Testosterone (n=31), age=36.5±12.5 Control (n=31), age=36.4±2.0	Case: transdermal testosterone with a daily single patch of 2.5 mg during the 5 days preceding ovarian stimulation Control: none	Cases: standard long downregulation protocol Controls: high dose gonadotrophins with a mini dose GnRH agonist protocol	Live birth rate Clinical pregnancy rate No. of oocytes Total dose of FSH Duration of FSH used Cancellation rate Adverse effects
Massin *et al* (14)	RCT	Inclusion: History of poor ovarian response to ovarian stimulation Evidence of a decreased ovarian reserve (D3 FSH >12 IU/l, E2 >70 pg/ml and inhibin B <45 pg/ml) Exclusion: Age >42 years History of previous ovarian surgery, ovarian endometriosis and endocrine and metabolic disorders	N=49 Testosterone (n=24), age=36.9±3.8 Control (n=25), age=37.3±4.0	Case: transdermal testosterone gel 1 g/day (10% absorption, 10 mg of testosterone) for 15–20 days preceding the ovarian stimulation Control: placebo	The same GnRH agonist protocol and the same starting dose of r FSH was used for both case and control group	Live birth rate Clinical pregnancy rate No. of oocytes Total dose of FSH Duration of FSH used Cancellation rate Adverse effects

COH, controlled ovarian hyperstimulation; RCT, randomized controlled trial; IVF/ICSI, *in vitro* fertilization/intracytoplasmic sperm injection; GnRH, gonadotropin-releasing hormone; FSH, follicle-stimulating hormone; D3, day 3 of menstrual cycle ; E2, estradiol; r-FSH, recombinant follicle-stimulating hormone.

**Table II:** Summary of study quality characteristics.

Study	Design	Level of evidence	Compliance	Testosterone protocol	Strengths	Weaknesses
Kim *et al* (21)	RCT N=110 Testosterone (n=55) Control (n=55) Recruitment from 2005 to 2009	2	100% completed	Case: transdermal testosterone gel Control: none	RCT High compliance Daily dose and duration of testosterone was determined on basis of previous study Ovarian stimulation protocol and initial gonadotropin dose were similar	Small sample size Sample size/power calculations not reported Lack of blinding to investigator Long time frame of study (4 years)
Fábregues *et al* (22)	RCT N=62 Testosterone (n=31) Control (n=31) Recruitment from 2006 to 2007	2	100% completed	Case: transdermal testosterone patch Control: none	RCT No drop-out Sample size/power calculated on basis of previous study Short time frame of study (10 months)	Not blinding design Down-regulation and ovarian stimulation protocols were different between the two groups Test power decreased because of higher than expected rate of patients undergoing ovum retrieval in control group
Massin *et al* (14)	RCT N=53 Testosterone (n=27) Control (n=26) Recruitment from 2001 to 2003	2	Drop-out rate 7.5%; n=53 recruited and randomized; results provided for n=49	Case: transdermal testosterone gel Control: placebo	RCT Double binding Down-regulation and ovarian stimulation protocol was comparable in the two groups	Small sample size Relatively high drop-out rate

RCT, randomized controlled trial.
